# Supplementary material for: Landscape topography structures the soil microbiome in arctic polygonal tundra
Source: Nat Commun. 2018 Feb 22;9:777. doi: 10.1038/s41467-018-03089-z (PMC5823929; doi:10.1038/s41467-018-03089-z)
Supplement: Supplementary file 1 — Supplementary Information [file 41467_2018_3089_MOESM1_ESM.pdf]

Landscape topography structures the soil microbiome in arctic polygonal tundra

Taş et al. 2018

Supplementary Table and Figures

Supplementary Tables

Supplementary Table 1. Soil and pore water chemistry for selected depths in deep cores from a flat-centered polygon. Values are averages of duplicate measurements per sample depth.

Depth (cm)	Horizon	pH	N (%)	C (%)	C/N	Nitrate (μM)	Nitrite (μM)	Sulfate (μM)	Iron (μM)	Cl (μM)	Na (μM)	Mg (μM)	Al (μM)	K (μM)	Ca (μM)
-2 to -7	organic	5.09	2.5	40.1	16.1	242.5	98.9	284.7	45.4	3.7E+02	2.9E+04	3.5E+03	5.0E+02	6.2E+03	8.8E+03
-15	organic	4.93	0.6	10.4	18.5	479.4	81.0	579.1	36.2	4.0E+02	5.1E+04	3.7E+03	1.1E+03	4.2E+02	8.2E+03
-25	mineral	4.54	1.2	18.7	15.8	60.4	75.6	302.3	42.4	3.1E+02	4.0E+04	1.6E+03	1.2E+03	1.9E+02	9.1E+03
-35	mineral	4.94	0.8	12.9	16.8	41.8	48.7	12.4	119.3	2.9E+02	5.0E+04	2.7E+03	1.2E+03	6.4E+02	7.2E+03
-50	permafrost	6.19	1.5	30.2	20.7	54.8	0.0	1.0	65.8	2.9E+03	2.5E+05	4.2E+04	1.8E+02	4.3E+03	4.4E+04
-65	permafrost	7.32	0.4	6.4	17.1	73.2	0.0	104.5	9.4	1.2E+04	1.1E+06	1.9E+05	9.5E+01	4.7E+04	1.4E+05
-80	permafrost	7.43	0.9	16.3	18.4	52.5	0.0	26.2	32.7	8.5E+03	7.7E+05	1.4E+05	6.6E+01	2.8E+04	9.5E+04
-95	permafrost	7.06	0.6	12.5	19.8	28.5	0.0	87.8	2.0	1.5E+04	1.1E+06	3.5E+05	5.0E+01	3.5E+04	2.2E+05
-115	permafrost	7.69	0.5	9.3	19.9	25.0	0.0	26.1	16.4	1.0E+04	7.5E+05	2.3E+05	8.2E+01	2.5E+04	1.2E+05
-125	permafrost	7.00	0.4	8.2	20.0	14.5	0.0	27.9	1.8	8.5E+03	6.2E+05	1.8E+05	1.2E+02	2.3E+04	8.8E+04
-140	permafrost	8.18	0.1	2.2	16.9	17.5	0.0	1108.0	0.7	3.1E+04	6.8E+05	7.4E+05	7.6E+01	1.1E+05	2.9E+05
-150	permafrost	7.69	0.1	2.3	16.7	12.2	22.7	1817.3	0.3	1.6E+04	1.2E+06	3.2E+05	1.3E+02	5.3E+04	1.3E+05
-165	permafrost	8.36	0.1	1.7	15.0	8.9	0.0	1717.9	0.5	4.5E+03	4.2E+05	7.2E+04	1.4E+02	3.0E+04	4.2E+04
-177	permafrost	8.48	0.2	2.5	16.0	11.4	0.0	4179.7	0.4	1.3E+04	1.1E+06	2.6E+05	1.8E+01	6.4E+04	1.3E+05
-192	permafrost	8.61	0.1	1.9	14.6	6.4	0.0	4752.2	0.2	1.2E+04	1.1E+06	2.3E+05	2.1E+01	5.4E+04	1.1E+05
-207	permafrost	8.57	0.2	2.6	15.4	0.0	0.0	4855.8	0.5	1.2E+04	1.0E+06	2.0E+05	1.0E+02	5.5E+04	1.1E+05
-217	permafrost	8.61	0.1	1.8	14.3	0.0	0.0	4036.9	0.3	7.7E+03	7.4E+05	1.3E+05	5.6E+01	5.0E+04	6.9E+04
-227	permafrost	8.58	0.1	2.1	15.1	0.0	0.0	7570.6	0.4	1.7E+04	1.4E+06	3.0E+05	7.9E+01	6.5E+04	1.6E+05
-237	permafrost	8.53	0.1	2.3	15.7	6.9	0.0	10126.7	0.3	2.5E+04	2.1E+06	4.0E+05	1.7E+01	9.0E+04	2.2E+05
-257	permafrost	8.70	0.1	1.8	12.5	0.0	0.0	20131.7	7.1	4.0E+04	4.1E+06	7.9E+05	9.6E+01	1.3E+05	3.7E+05
-265	permafrost	8.37	0.1	1.8	12.4	36.7	0.0	22947.8	1.1	4.6E+04	4.6E+06	1.0E+06	9.0E+01	1.6E+05	4.5E+05

Supplementary Table 2. Active layer samples from different polygon types and futures were used in metagenomics analysis. Samples were sequences with Illumina HiSeq 2000 platform and resulted in variable number of high quality reads.

**Polygon type**	**Distance (m)**	**Soil horizon**	**Number of reads**
High Centered	47	Organic	9.5E+07
	47	Mineral	9.6E+06
	50	Organic	5.5E+06
	50	Mineral	1.4E+08
	53	Organic	1.5E+08
	53	Mineral	2.9E+08
Flat Centered	204	Organic	2.1E+08
	210	Organic	2.6E+08
	210	Mineral	1.2E+08
	214	Organic	1.1E+07
	214	Mineral	4.9E+07
	405	Organic	4.5E+07
Low Centered	405	Mineral	3.1E+08
	408.5	Organic	1.6E+08
	408.5	Mineral	7.0E+05
	411	Organic	2.8E+08
	411	Mineral	2.8E+08
	415	Organic	1.9E+08

Supplementary Table 3. 16S rRNA gene based alpha-diversity metrics from active layer samples of different polygon types

Polygon Type	Soil horizon	Distance (m)	Faith's PD	Chao1	Shannon H'
High Centered	Organic	47	124	8392	11.4
	Mineral	47	118	8099	11.2
	Organic	48	128	8273	11.3
	Mineral	48	126	8056	11.3
	Organic	50	123	7435	11.2
	Mineral	50	107	7273	10.8
	Organic	53	104	6691	10.9
	Organic	53	119	7583	11.1
	Mineral	53	122	7714	11.1
	Mineral	53	99	6529	10.9
	Organic	58	130	8698	11.3
	Mineral	58	115	7678	11.1
Flat Centered	Organic	204.5	112	8221	10.8
	Mineral	204.5	96	5536	10.6
	Mineral	205	86	5920	10.1
	Organic	207	130	9415	11.4
	Mineral	207	85	6081	10.1
	Organic	210	84	5677	9.9
	Mineral	210	107	6855	11.0
	Organic	214	126	8526	11.4
	Mineral	214	98	7425	10.3
Low Centered	Organic	405	103	6546	10.9
	Mineral	405	91	4801	10.3
	Organic	406	104	7204	10.8
	Mineral	406	72	4477	9.8
	Organic	408.5	100	4935	9.9
	Mineral	408.5	65	3807	9.6
	Organic	411	95	5517	10.5
	Mineral	411	75	4401	9.9
	Mineral	411	88	5203	10.4
	Organic	416	97	5090	10.6

Supplementary Table 4. Pearson coefficients of significant ($p<0.05$) correlations between soil chemistry and relative abundances of major prokaryotic phyla in active layer samples. Not significant (n.s.) values are not reported.

	**Depth (cm)**	**Moisture**	**C/N**	**Total C (%)**	**Total N (%)**	**Organic Carbon**	**Conductivity (uS/cm)**	**pH**
Actinobacteria	0.40	n.s.	n.s.	-0.40	-0.39	n.s.	n.s.	n.s.
Alphaproteobacteria	n.s.	n.s.	n.s.	n.s.	n.s.	n.s.	n.s.	n.s.
Acidobacteria	-0.52	n.s.	n.s.	n.s.	n.s.	n.s.	n.s.	n.s.
Betaproteobacteria	n.s.	n.s.	n.s.	n.s.	n.s.	n.s.	n.s.	0.39
Verrucomicrobia	n.s.	n.s.	n.s.	n.s.	n.s.	n.s.	n.s.	n.s.
Bacteroidetes	n.s.	n.s.	n.s.	n.s.	n.s.	0.43	n.s.	n.s.
Deltaproteobacteria	n.s.	0.62	n.s.	n.s.	n.s.	n.s.	n.s.	n.s.
Gammaproteobacteria	n.s.	n.s.	n.s.	n.s.	n.s.	n.s.	n.s.	n.s.
Chloroflexi	n.s.	n.s.	n.s.	n.s.	n.s.	n.s.	n.s.	n.s.
Planctomycetes	n.s.	n.s.	n.s.	n.s.	n.s.	n.s.	n.s.	n.s.
Gemmatimonadetes	-0.38	n.s.	n.s.	0.51	0.50	n.s.	n.s.	n.s.
Cyanobacteria	n.s.	n.s.	n.s.	n.s.	n.s.	n.s.	n.s.	n.s.
WPS-2	n.s.	n.s.	n.s.	n.s.	n.s.	n.s.	n.s.	n.s.
Firmicutes	n.s.	0.36	n.s.	n.s.	n.s.	0.39	n.s.	n.s.
AD3	n.s.	n.s.	n.s.	n.s.	n.s.	n.s.	n.s.	n.s.
Euryarchaeota	n.s.	0.48	n.s.	n.s.	n.s.	n.s.	n.s.	n.s.
Caldiserica	n.s.	0.61	n.s.	n.s.	n.s.	0.46	n.s.	n.s.
Elusimicrobia	-0.54	n.s.	n.s.	n.s.	n.s.	n.s.	0.43	0.39
Armatimonadetes	-0.41	n.s.	n.s.	n.s.	n.s.	n.s.	n.s.	n.s.
Fibrobacteres	-0.50	n.s.	n.s.	0.43	0.48	n.s.	n.s.	n.s.
OD1	n.s.	n.s.	n.s.	n.s.	n.s.	n.s.	n.s.	n.s.
Nitrospirae	n.s.	n.s.	n.s.	n.s.	n.s.	n.s.	n.s.	n.s.
Chlorobi	n.s.	n.s.	n.s.	n.s.	n.s.	n.s.	n.s.	n.s.
WYO	n.s.	n.s.	n.s.	n.s.	n.s.	n.s.	n.s.	n.s.
Crenarchaeota	n.s.	n.s.	n.s.	n.s.	n.s.	n.s.	n.s.	n.s.

Supplementary Table 5. Bin identification, completeness and contamination via CheckM analysis

Bin Id	Polygon	Soil Horizon	Marker lineage	Genome Size (bp)	% abundance in metagenome	GC content (%)	0	1	2	3+	Completeness (%)	Contamination (%)	Strain heterogeneity
53.003.002	HC	Organic	k_Bacteria	4.5E+06	1.22	59	6	177	8	0	95.6	4.8	0.0
53.005	HC	Organic	p_Bacteroidetes	5.4E+06	1.21	40	17	285	14	1	97.3	2.2	20.0
53-4.002	HC	Mineral	k_Bacteria	4.1E+06	2.69	68	6	154	3	0	95.9	2.7	66.7
53-4.004	HC	Mineral	c_Alphaproteobacteria	4.6E+06	2.14	66	5	325	17	2	98.3	2.7	13.0
53-4.005	HC	Mineral	p_Bacteroidetes	4.0E+06	1.12	36	4	304	9	0	98.1	3.3	11.1
53-4.010	HC	Mineral	o_Actinomycetales	3.3E+06	0.79	66	10	287	17	1	96.9	4.3	15.0
53-4.014	HC	Mineral	p_Bacteroidetes	4.6E+06	0.65	41	2	292	10	0	99.0	1.9	0.0
53-4.027	HC	Mineral	p_Bacteroidetes	4.5E+06	0.46	36	1	291	24	1	99.5	7.5	11.1

Bin Id	Polygon	Soil Horizon	Marker lineage	Genome Size (bp)	% abundance in metagenome	GC content (%)	0	1	2	3+	Completeness (%)	Contamination (%)	Strain heterogeneity
204.018	FC	Organic	k_Bacteria	4.4E+06	0.80	39	8	78	16	2	89.0	10.1	68.2
210.008	FC	Organic	p_Actinobacteria	3.4E+06	1.35	69	17	182	7	0	91.6	3.1	0.0
210.014	FC	Organic	k_Bacteria	3.4E+06	0.79	68	8	167	14	2	94.4	7.2	10.0
210.017	FC	Organic	k_Bacteria	6.0E+06	1.12	62	3	158	24	6	97.5	8.6	4.8
210.021	FC	Organic	k_Bacteria	3.9E+06	0.60	57	4	212	10	5	97.7	13.8	11.4
210.022	FC	Organic	k_Bacteria	2.1E+06	0.35	60	2	73	26	3	98.1	13.7	57.9
214-4.002	FC	Mineral	o_Actinomycetales	5.6E+06	5.96	66	13	275	20	2	94.9	7.5	22.2
214-4.005	FC	Mineral	k_Bacteria	2.2E+06	1.03	60	4	161	6	0	97.0	1.1	33.3

Bin Id	Polygon	Soil Horizon	Marker lineage	Genome Size (bp)	% abundance in metagenome	GC content (%)	0	1	2	3+	Completeness (%)	Contamination (%)	Strain heterogeneity
C-5.032	LC	Mineral	p_Bacteroidetes	3.8E+06	0.50	36	14	258	31	1	94.2	12.4	14.7
D.003	LC	Organic	c_Alphaproteobacteria	4.3E+06	1.52	65	24	315	7	3	93.7	3.1	25.0
D.006	LC	Organic	k_Bacteria	7.6E+06	1.95	45	3	65	15	21	96.6	15.0	86.9
D.018	LC	Organic	p_Bacteroidetes	5.1E+06	0.46	41	8	294	12	3	97.2	5.7	0.0
D.019	LC	Organic	f_Xanthomonadaceae	3.5E+06	0.33	58	23	608	28	1	95.2	3.5	9.7
D.030	LC	Organic	p_Bacteroidetes	3.9E+06	0.25	52	16	276	12	0	93.8	4.5	16.7
D-3.007	LC	Mineral	k_Bacteria	3.1E+06	1.86	62	12	142	12	5	90.6	8.3	18.5
D-3.010	LC	Mineral	k_Bacteria	4.6E+06	1.57	61	6	177	6	2	95.6	6.8	8.3
D-3.013	LC	Mineral	k_Bacteria	4.1E+06	0.87	48	0	145	3	0	100.0	2.2	66.7
D-3.020	LC	Mineral	c_Alphaproteobacteria	2.7E+06	0.64	60	24	313	12	0	90.7	3.2	33.3
D-3.025	LC	Mineral	c_Deltaproteobacteria	6.4E+06	0.94	64	34	208	5	1	90.0	4.0	12.5
D-3.026	LC	Mineral	p_Actinobacteria	2.5E+06	0.67	69	16	172	17	1	89.3	7.4	0.0
D-3.034	LC	Mineral	o_Actinomycetales	3.6E+06	0.55	62	23	290	2	0	94.1	1.1	0.0
D-3.042	LC	Mineral	k_Bacteria	2.8E+06	0.34	57	9	180	2	0	96.3	1.7	0.0
D-3.052	LC	Mineral	p_Bacteroidetes	5.0E+06	0.39	38	29	279	8	1	93.5	3.6	18.2
D-3.054	LC	Mineral	c_Alphaproteobacteria	2.6E+06	0.25	54	30	309	10	0	94.3	2.7	10.0
E.030	LC	Organic	k_Bacteria	3.4E+06	0.45	68	18	153	19	1	91.3	12.9	4.6
E.001	LC	Organic	p_Euryarchaeota	1.3E+06	0.92	52	135	71	20	3	41.1	9.1	10.3

Supplementary Table 6. 16S rRNA genes identified in genomic bins

Bin Id	Polygon	16S rRNA gene (bp)	Organism	Max score	Total score	Query coverage	E value	Identity
53.003.002	HC	NA						
53.005	HC	1418	Arcticibacter svalbardensis strain MN12-7	1386	1386	99%	0	85%
53-4.002	HC	NA						
53-4.004	HC	1452	Methylocella silvestris strain BL2	1908	1908	91%	0	93%
53-4.005	HC	1051	Solitalea canadensis strain DSM 3403	1208	1208	92%	0	89%
53-4.010	HC	NA						
53-4.014	HC	NA						
53-4.027	HC	NA						
204.018	FC	NA						
210.008	FC	1498	Conexibacter woesei strain DSM 14684	2342	2342	100%	0	95%
210.014	FC	1438	Geobacter metallireducens strain GS-15	1339	1339	99%	0	84%
210.017	FC	1176	Thermoanaerobaculum aquaticum strain MP-01	1373	1373	100%	0	88%
210.021	FC	NA						
210.022	FC	NA						
214-4.002	FC	1249	Frankia alni ACN14a strain ACN14A	1847	1847	99%	0	93%
214-4.005	FC	1525	Aciditerrimonas ferrireducens strain IC-180	1875	1875	96%	0	90%
C-5.032	LC	1107	Chitinophaga niabensis strain JS13-10	1546	1546	95%	0	93%
D.003	LC	1445	Devosia geojensis strain BD-c194	1982	1982	100%	0	91%
D.006	LC	NA						
D.018	LC	NA						
D.019	LC	1539	Rhodanobacter umsongensis strain GR24-2	2403	2403	87%	0	99%
D.030	LC	1484	Chitinophaga eiseniae strain YC6729	1882	1882	100%	0	90%
D-3.007	LC	NA						
D-3.010	LC	1558	Haliangium ochraceum DSM 14365	1784	3569	100%	0	87%
D-3.013	LC	1584	Geobacter metallireducens strain GS-15	1280	1280	97%	0	82%
D-3.020	LC	NA						
D-3.025	LC	NA						
D-3.026	LC	NA						
D-3.034	LC	NA						
D-3.042	LC	1488	Acidipila rosea strain AP8	1903	1903	94%	0	91%
D-3.052	LC	NA						
D-3.054	LC	NA						
E.030	LC	974	Pelobacter carbinolicus strain DSM 2380	913	913	99%	0	84%
E.001	LC	1441	Methanolinea tarda strain NOBI-1	1591	1591	99%	0	87%

Supplementary Table 7. Identification of bin phylogeny based on *dnaG*, *frr*, *infC*, *nusA*, *pyrG*, *pgk*, *rpl*, *rpm*, *rpo*, *rps*, *tsf* and *smpB* gene identification with Amphora2

Bin Id	Polygon	Soil Horizon	Phylum
53.003.002	HC	Organic	Acidobacteria
53-4.002	HC	Mineral	Bacteria (kingdom)
204.018	FC	Organic	Bacteroidetes
210.014	FC	Organic	Acidobacteria
210.017	FC	Organic	Acidobacteria
210.021	FC	Organic	Verrucomicrobia
210.022	FC	Organic	Actinobacteria
D.006	LC	Organic	Bacteroidetes
D-3.007	LC	Mineral	Actinobacteria
D-3.010	LC	Mineral	Acidobacteria
D-3.013	LC	Mineral	Bacteria (kingdom)
E.030	LC	Organic	Bacteria (kingdom)

Supplementary Table 8. Gravimetric soil moisture as reported by Hubbard *et al* 2013

**Polygon type**	**Distance (m)**	**Soil horizon**	**Gravimetric Soil Moisture (g gr soil-1)**
High Centered	47	Organic	0.74
	47	Mineral	0.58
	48	Organic	0.72
	48	Mineral	0.66
	50	Organic	0.91
	50	Mineral	0.60
	52	Organic	0.66
	52	Mineral	0.61
	53	Organic	0.64
	53	Mineral	0.58
	54	Organic	0.61
	54	Mineral	0.58
	58	Organic	0.67
	58	Mineral	0.50
Flat Centered	204.5	Organic	0.86
	204.5	Mineral	0.51
	207	Organic	0.73
	207	Mineral	0.51
	210	Organic	0.7
	210	Mineral	0.24
	214	Organic	0.75
	214	Mineral	0.37
Low Centered	405	Organic	0.79
	405	Mineral	0.44
	406	Organic	0.82
	406	Mineral	0.49
	408.5	Organic	0.85
	408.5	Mineral	0.55
	411	Organic	0.91
	411	Mineral	0.54
	415	Organic	0.89

Supplementary Figures

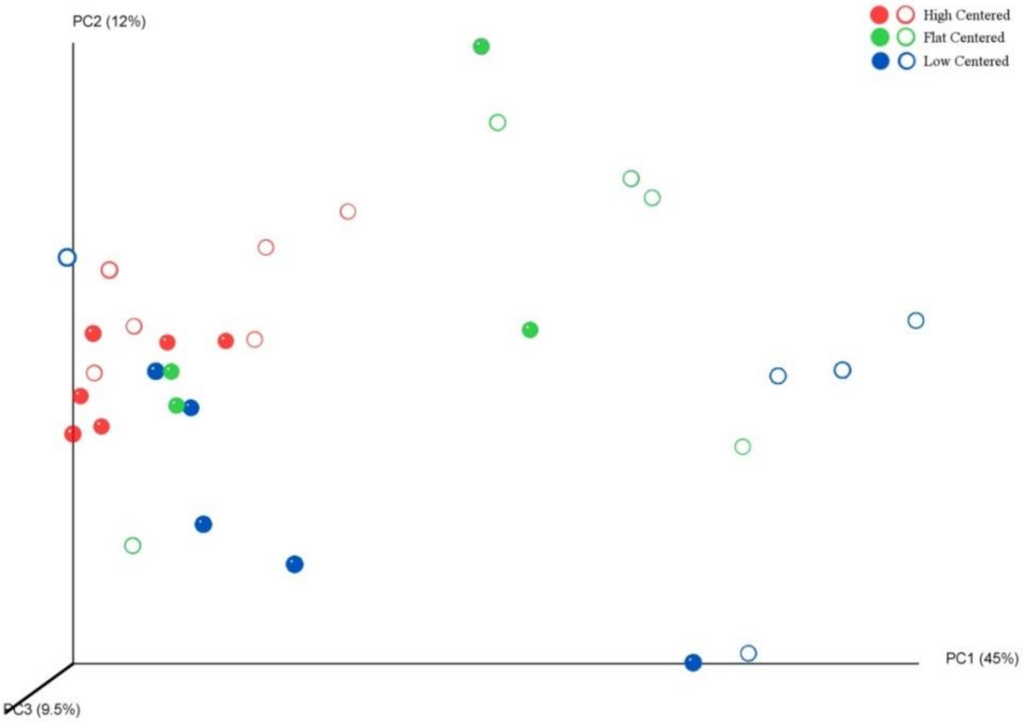

Supplementary Figure 1. Unifrac analysis of 16S rRNA sequence variation within and between polygons. Organic (closed) and mineral (open) soil depths were represented as circles. The variance explained by each principal component (PC) axis is given in parentheses. Effects of main factors on prokaryotic beta-diversity similarity were assessed by analysis of similarity (ANOSIM). Main factors represent polygon type (HC, FC and LC; ANOSIM R=0.432, p<0.001) and soil horizon (organic and mineral; (ANOSIM R=0.117, p=0.044). Additionally, Adonis analysis resulted in significant correlations between organic carbon content (F=2.02, r^2=0.057, p= 0.032) and total nitrogen (F=2.33, r^2=0.065, p=0.019) of the samples and observed clustering.

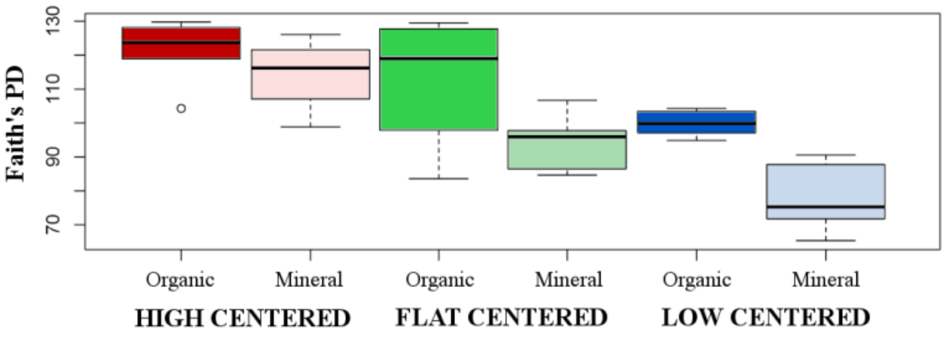

Supplementary Figure 2. Phylogenetic alpha diversity (Faith's PD) were significantly different amongst different polygons (F=18.64, p<0.001) and soil horizons (F=14.34, p<0.001). Between different polygon types and soil horizons Faith's PD was higher in high centered polygons. Within each polygon no significant differences were observed between Faith's PD of soil horizons (F=1.34, p=0.279). Error bars represent the standard error.

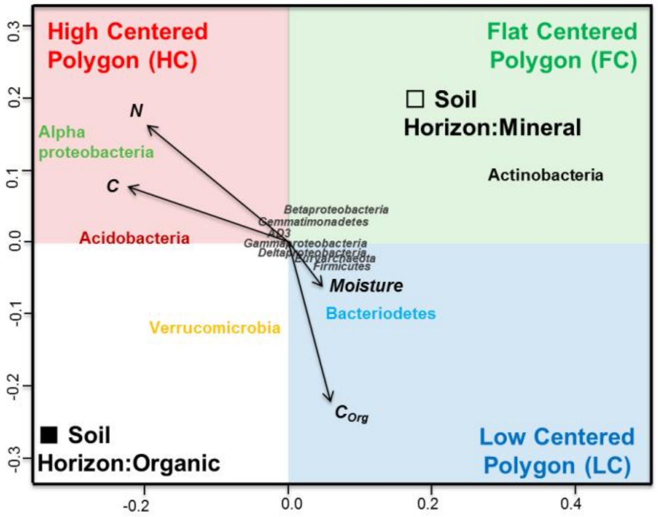

	Df	ChiSquare	F	p
Total C	1	0.035	3.489	0.003
Polygon Type	2	0.060	2.998	0.003
Organic C	1	0.027	2.756	0.020
Soil Horizon	1	0.024	2.465	0.031
pH	1	0.023	2.284	0.039
Total N	1	0.014	1.386	0.209
Moisture	1	0.013	1.330	0.216
Residual	21	0.209		

Supplementary Figure 3. Canonical component analysis explained 55% of the observed variation (p=0.005) in microbial community abundances of different polygons.

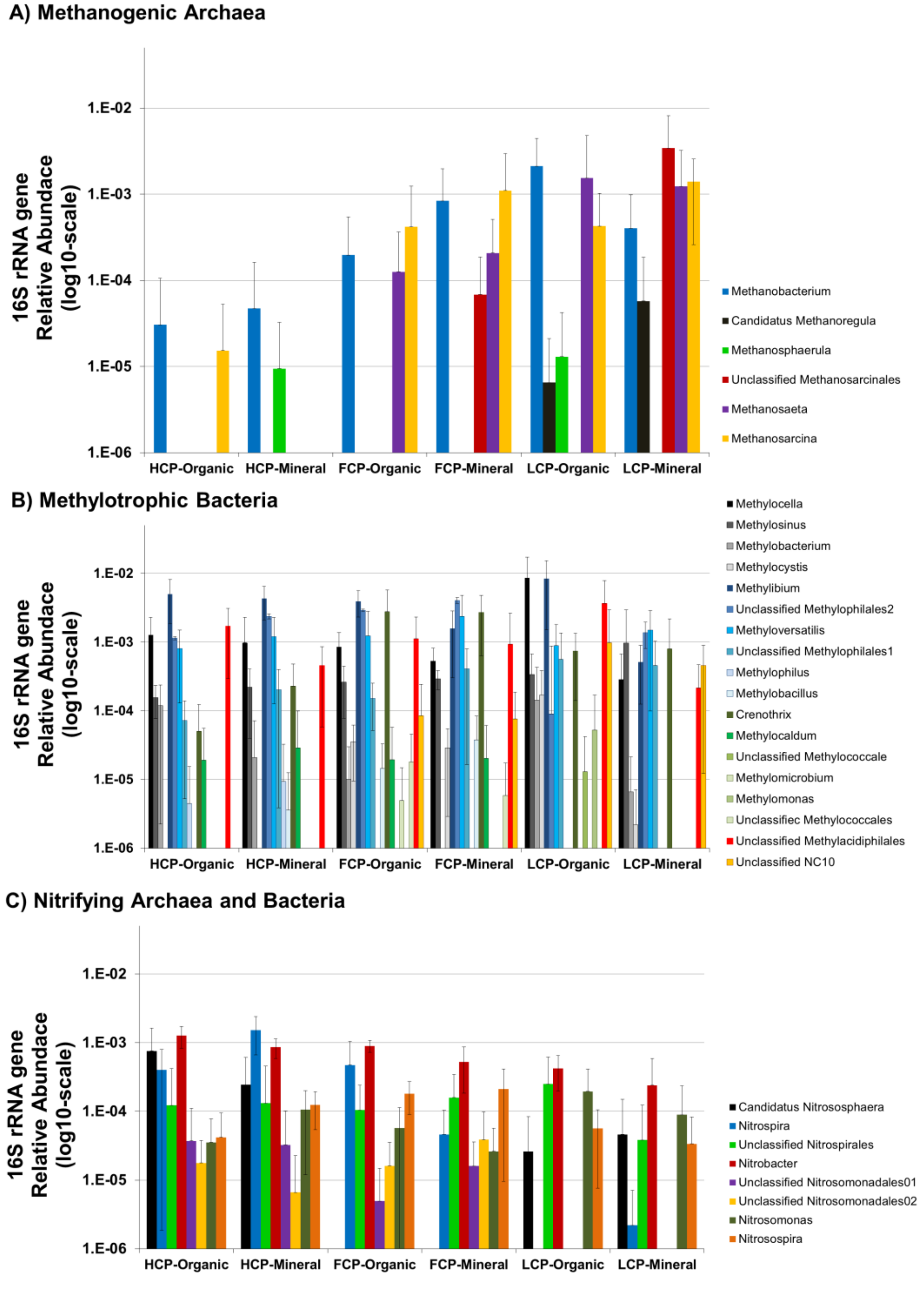

Supplementary Figure 4. 16S rRNA sequence based distribution of A) methanogenic Archaea B) methane oxidizing Bacteria and C) nitrifying Bacteria and Archaea at genus level classification in Barrow polygons. Error bars represent the standard error.

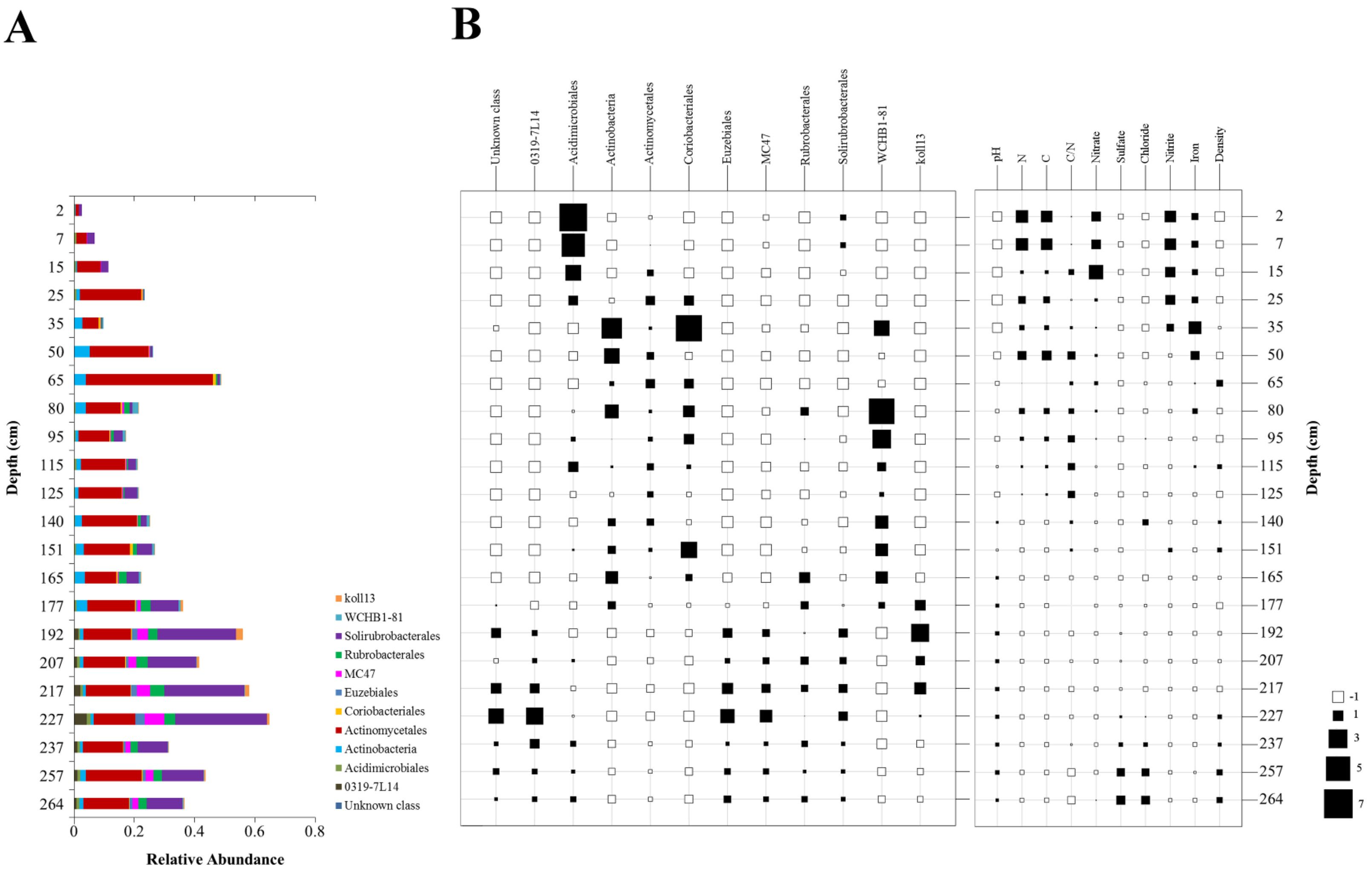
A
B
Depth (cm)
Relative Abundance
0
0.2
0.4
0.6
0.8
2
7
15
25
35
50
65
80
95
115
125
140
151
165
177
192
207
217
227
237
257
264
koll13
WCHB1-81
Solirubrobacterales
Rubrobacterales
MC47
Euzebiales
Coriobacteriales
Actinomycetales
Actinobacteria
Acidimicrobiales
0319-7L14
Unknown class
pH
N
C
C/N
Nitrate
Sulfate
Chloride
Nitrite
Iron
Density
-1
1
3
5
7

Supplementary Figure 5. A) Order level distribution of Actinobacterial populations along the depth gradient from the deep cores of a flat-centered polygon. Actinomycetales and Solirubrobacterales were the most abundant orders. B) Canonical correlations amongst relative abundances of Actinobacterial orders and sample chemistry at different depths. Size of squares corresponds to the strength of positive (black) or negative (white) correlations. Most Actinobacterial orders except Acidimicrobiales and Solirubrobacteriales were negatively correlated with high C, N, iron, nitrate and nitrate content in the organic active layer soils. Actinomycetales and Coribacteriales was positively correlated with high iron levels in mineral soils. In permafrost, strongest positive correlations amongst Actinobacterial orders and all sample chemistry was with pH.

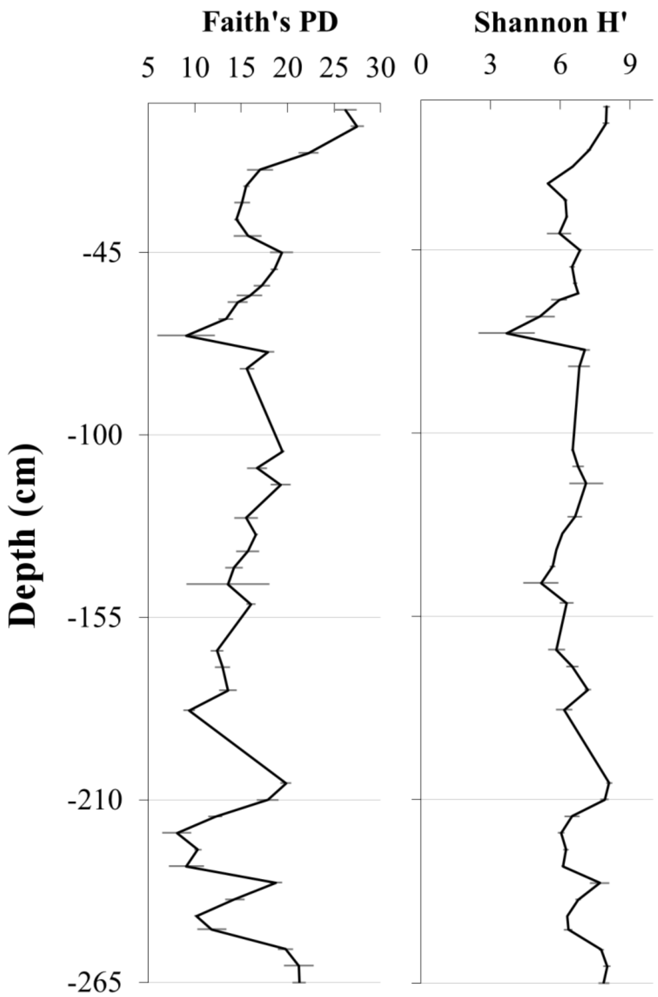

Supplementary Figure 6. Phylogenetic alpha diversity (Faith's PD and Shannon H') changed drastically along the depth profile. Error bars represent the standard deviation observed between duplicate cores and sub-samples in each depth (per depth n=8 between 0 to 1 m, n=4 between 1 to 2.65m).

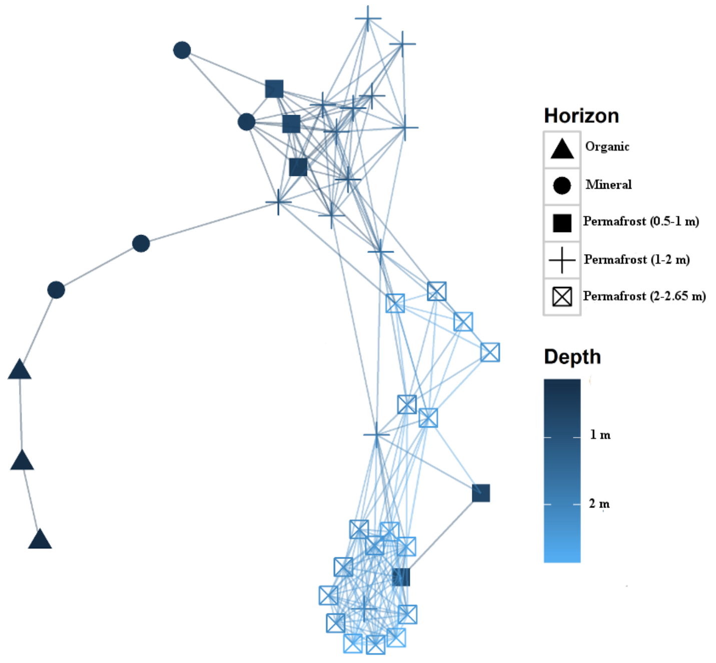

Supplementary Figure 7. Network diagram of deep core samples (nodes) that are linked if they are within the specified Bray distance. Symbols correspond to different soil horizons at different depths. Colors correspond to shallow (dark blue) to deep (light blue) sampling depths. The Bray distance is determined by pooling the OTUs from the replicates of each depth interval (n=8 between 0 to 1 m, n=4 between 1 to 2.65m) and representing their relatedness as the ratio of shared to total OTUs. Clustering patterns broadly corresponded to the changes in depth intervals. Interestingly there were no clusters amongst organic and mineral horizons of the active layer samples. Permafrost samples showed stronger clustering patterns with increasing depth.

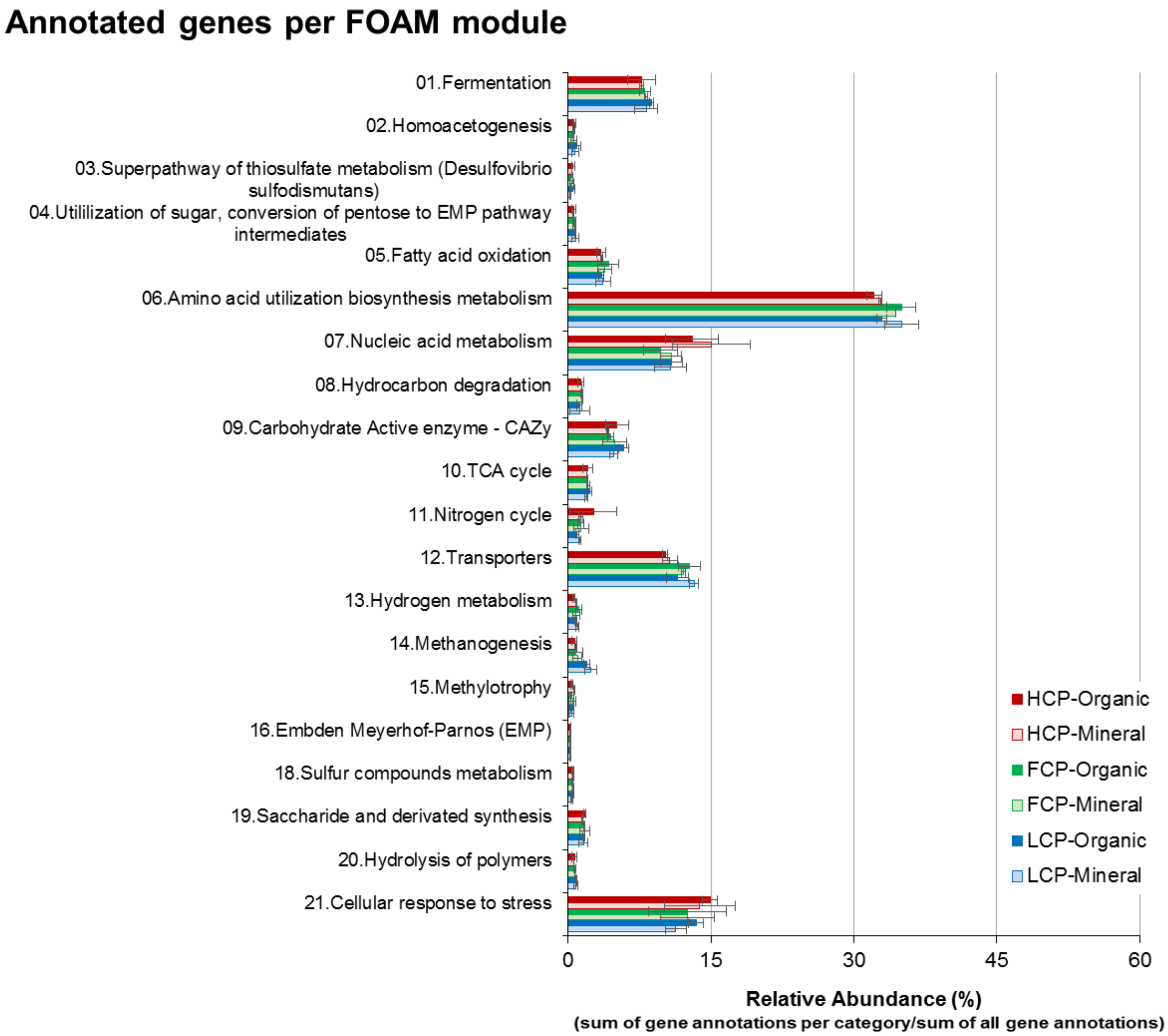

Supplementary Figure 8. Changes in the relative gene abundances in Barrow metagenomes based on FOAM module classifications. Error bars represent the standard error.

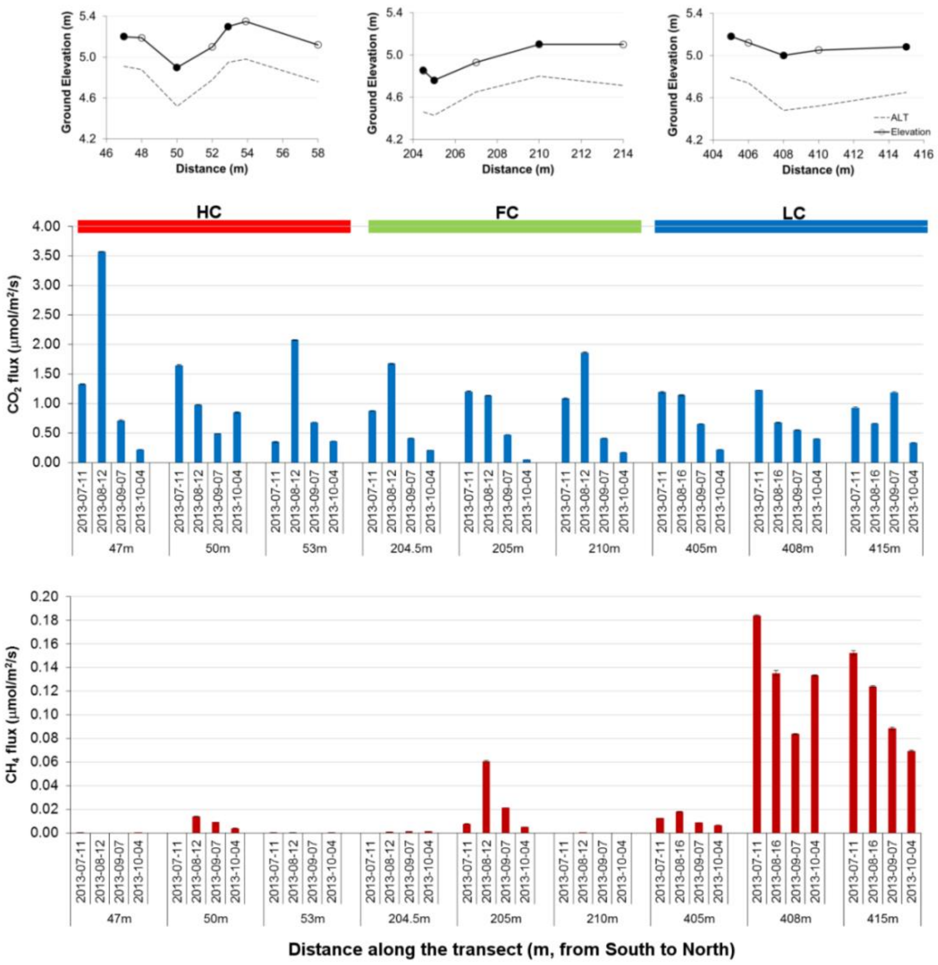

Supplementary Figure 9. CO_2 and CH_4 fluxes measurements collected in 2013 from high-centered (HC, red bar), flat-centered (FC, green bar) and low-centered (LC, blue bar) polygons across the transect[9]. Circles show sampling locations for metagenomes. Closed circles (●) shows the sampling points in each polygon (n=3 per polygon) for CO_2 and CH_4 fluxes measurements. When present, error bars show the standard error for fluxes from 3-4 replicate chambers within the same polygon feature. Details for 2013 measurements are provided in Materials and Methods. LC polygons were consistent sources of CH_4 throughout snow-free season. In HC and FC polygons, however, only wet areas such as troughs had detectable CH_4 fluxes, which were 12-40 times less ($F=21.09$, $p=4.549e-07$) than those from LC polygons.

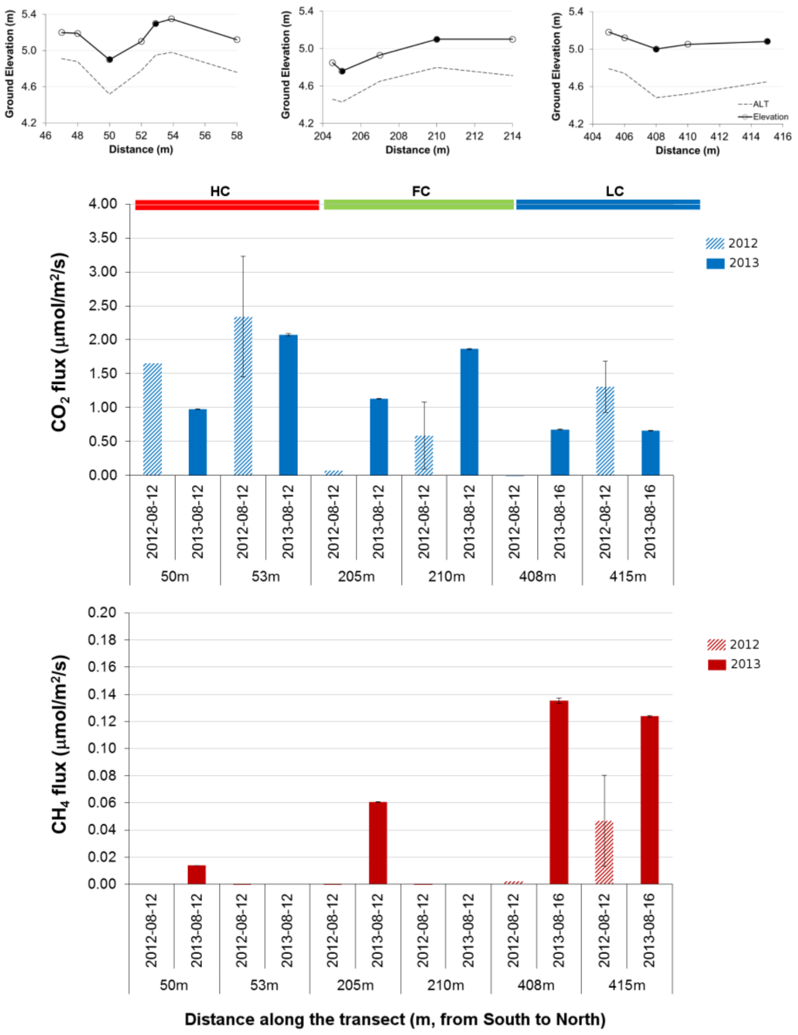

Supplementary Figure 10. Comparison between CO_2 and CH_4 flux measurements collected in August 2012 and 2013 from high-centered (HC, red bar), flat-centered (FC, green bar) and low-centered (LC, blue bar) polygons across the transect[9]. Closed circles (●) shows the sampling points in each polygon (n=2 per polygon) for CO_2 and CH_4 fluxes measurements. In 2012, fluxes were measured from three replicate locations in polygon centers and one location in polygon troughs in HC and FC polygons and a polygon rim in LC polygons. When present, error bars show the standard deviations for the replicate measurement locations in polygon centers. Details for 2013 measurements are provided in Supplementary Figure 9 and Materials and Methods.

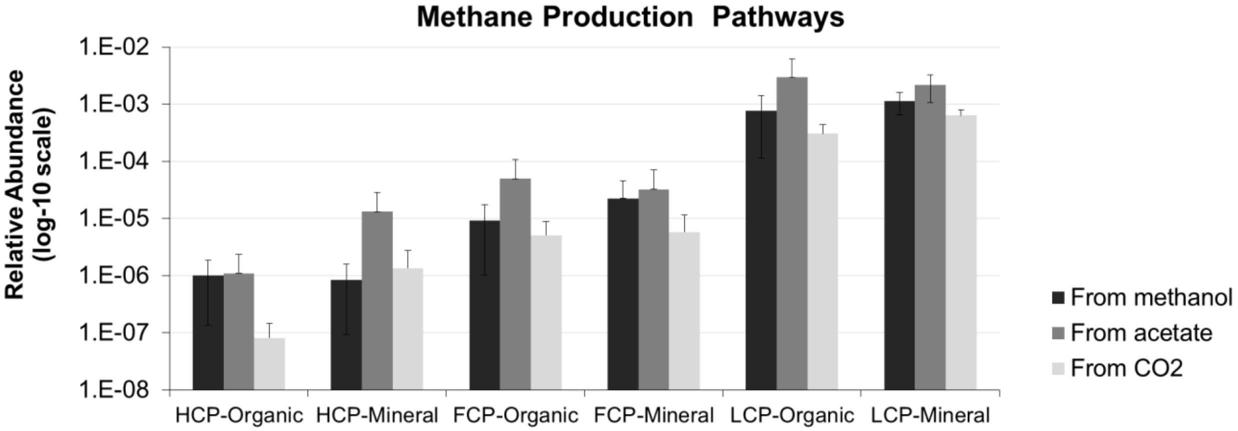

Supplementary Figure 11. Changes in the relative gene abundances of methane production pathways in Barrow metagenomes calculated from detected functional genes. Methanol pathway includes: *methanol-5-hydroxybenzimidazolylcobamide Co-methyltransferase* [EC:2.1.1.90] (*mtaB*) and *methanol corrinoid protein* (*mtaC*). Acetate pathway includes: *phosphate acetyltransferase* [EC:2.3.1.8] (*pta*), *acetyl-CoA synthetase* [EC:6.2.1.1] (*ACSS*), *acetyl-CoA decarbonylase/synthase complex subunit delta* [EC:2.1.1.245] (*cdhD*), *acetyl-CoA decarbonylase/synthase complex subunit epsilon* (*cdhB*), *tetrahydromethanopterin S-methyltransferase* [EC:2.1.1.86] subunit A,B,C,D,E, F (*mtrA, mtrB, mtrC, mtrD, mtrE, mtrF*). CO_2 pathway includes: *4Fe-4S ferredoxin* (*fwdF*), *formylmethanofuran dehydrogenase* [EC:1.2.99.5] subunit A, B,C,D (*fwdA, fwdB, fwdC, fwdD*), *4Fe-4S ferredoxin* (*fwdG*), *4Fe-4S ferredoxin (fwdH), methylenetetrahydromethanopterin dehydrogenase* [EC:1.5.98.1] (*mtd*). Error bars represent the standard error.

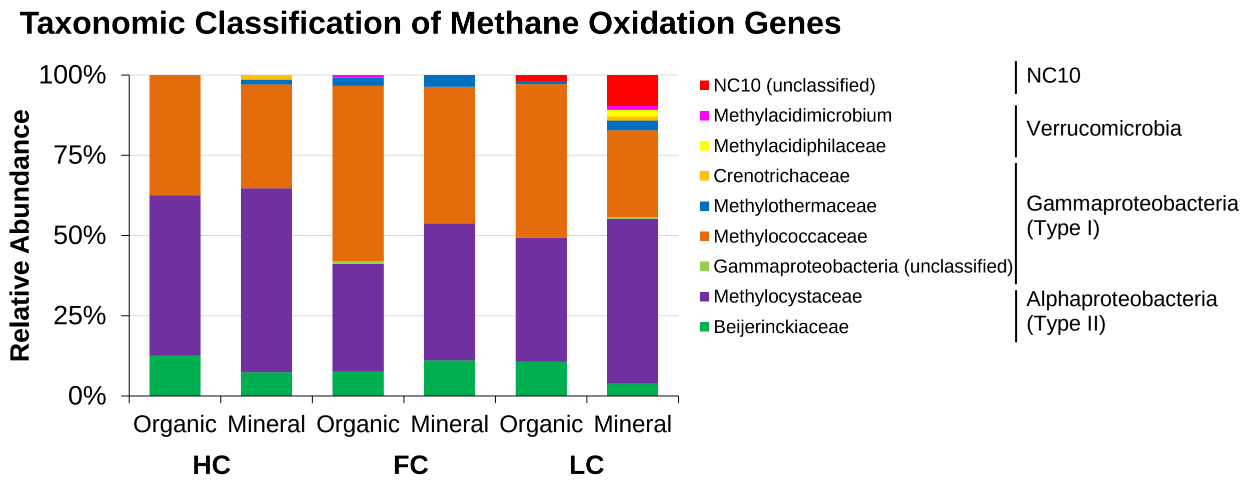

Supplementary Figure 12. Taxonomic distribution (rank order: Family) of particulate methane monooxygenase (pmoABC) and soluble methane monooxygenase (mmoXYZ) genes in Barrow metagenomes.

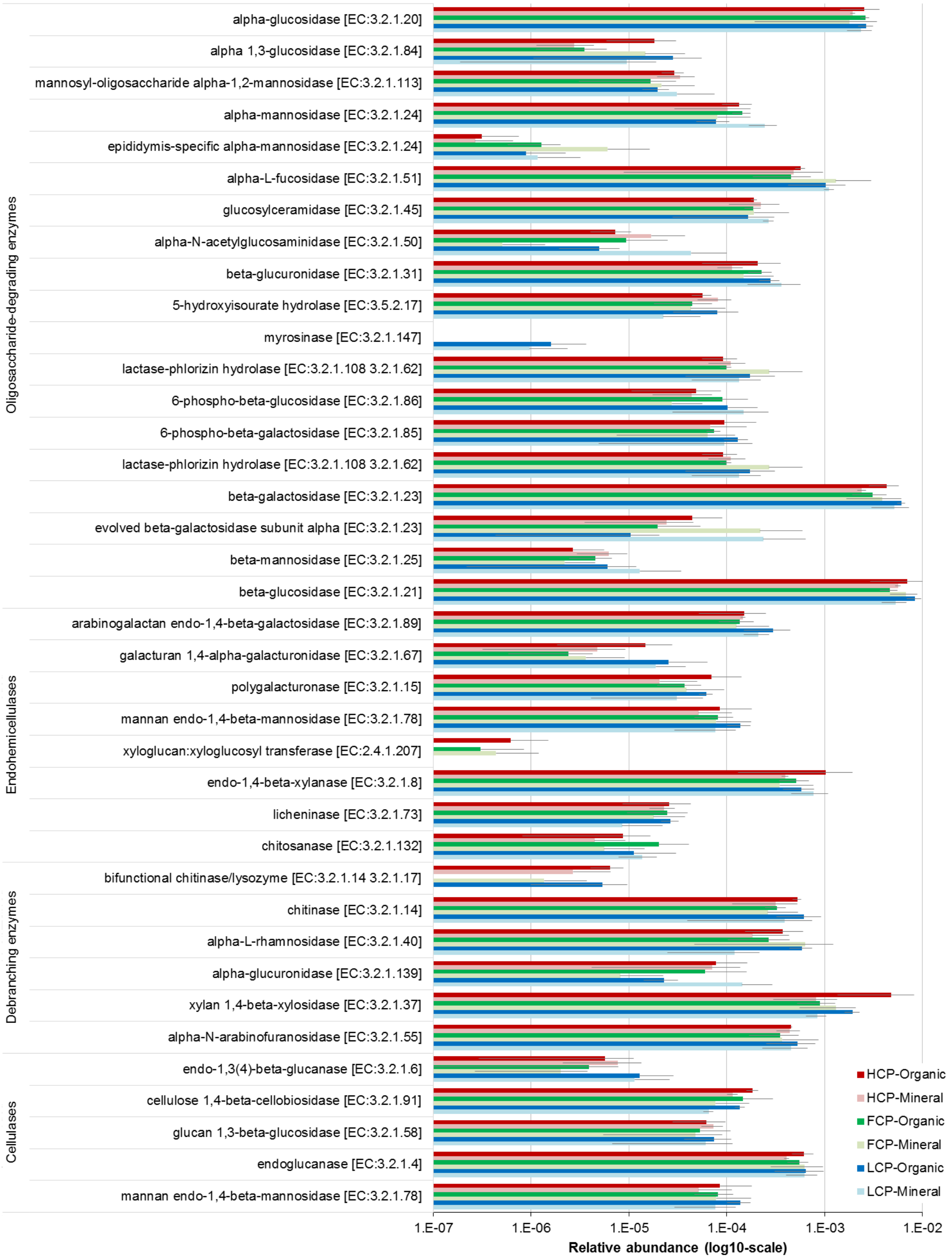

Supplementary Figure 13. Relative distribution of carbohydrate-active enzymes (CAZymes) in Barrow metagenomes. Error bars represent the standard error.

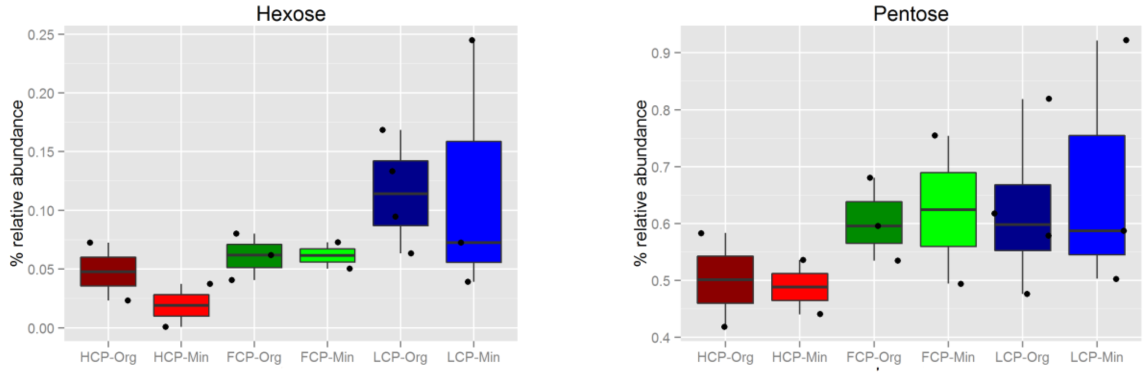

Supplementary Figure 14. Relative distribution of genes involved in sugar utilization in Barrow metagenomes were marginally but significantly different for Hexose degradation (F=4.18, p= 0.038) but not for Pentose degradation. Error bars represent the standard error.

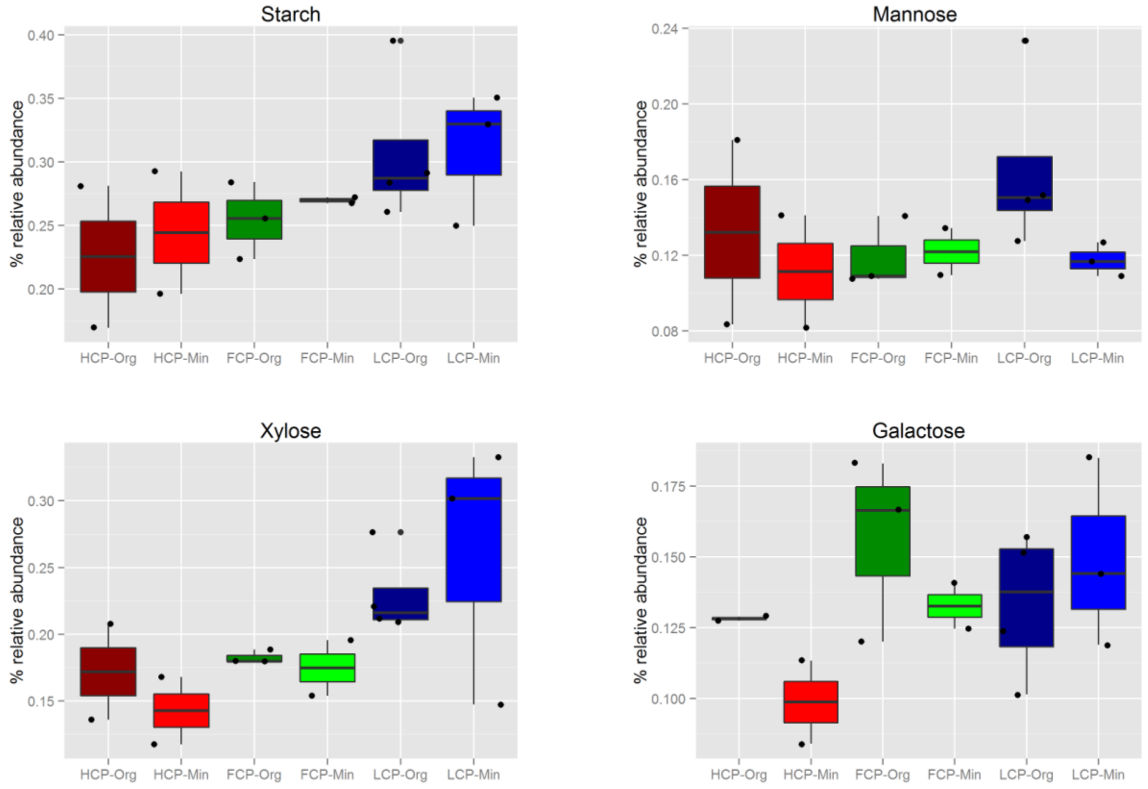

Supplementary Figure 15. Relative distribution of genes involved in polymer hydrolysis in Barrow metagenomes. LC polygons contained higher Xylose degradation genes than HC and FC polygons (*rho*=0.61, p=0.006). Error bars represent the standard error.

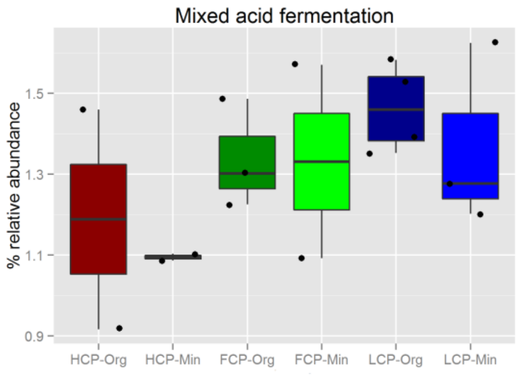

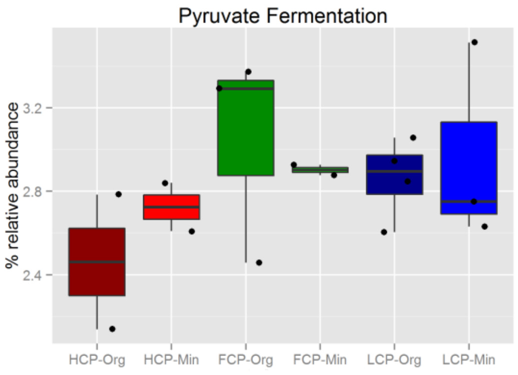

Supplementary Figure 16. Relative distribution of genes involved in fermentation in Barrow metagenomes. Mixed acid fermentation genes were found in higher abundance in LC polygons (*rho*=0.44, p=0.041) than in HC polygons. Error bars represent the standard error.

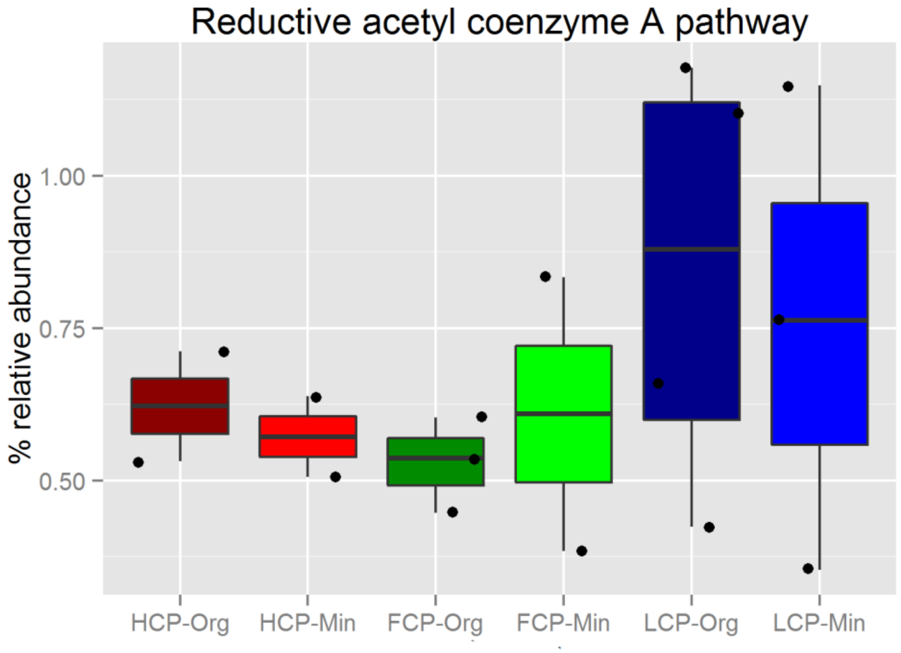

Supplementary Figure 17. Relative distribution of genes involved in homoacetogenesis in Barrow metagenomes. Error bars represent the standard error.

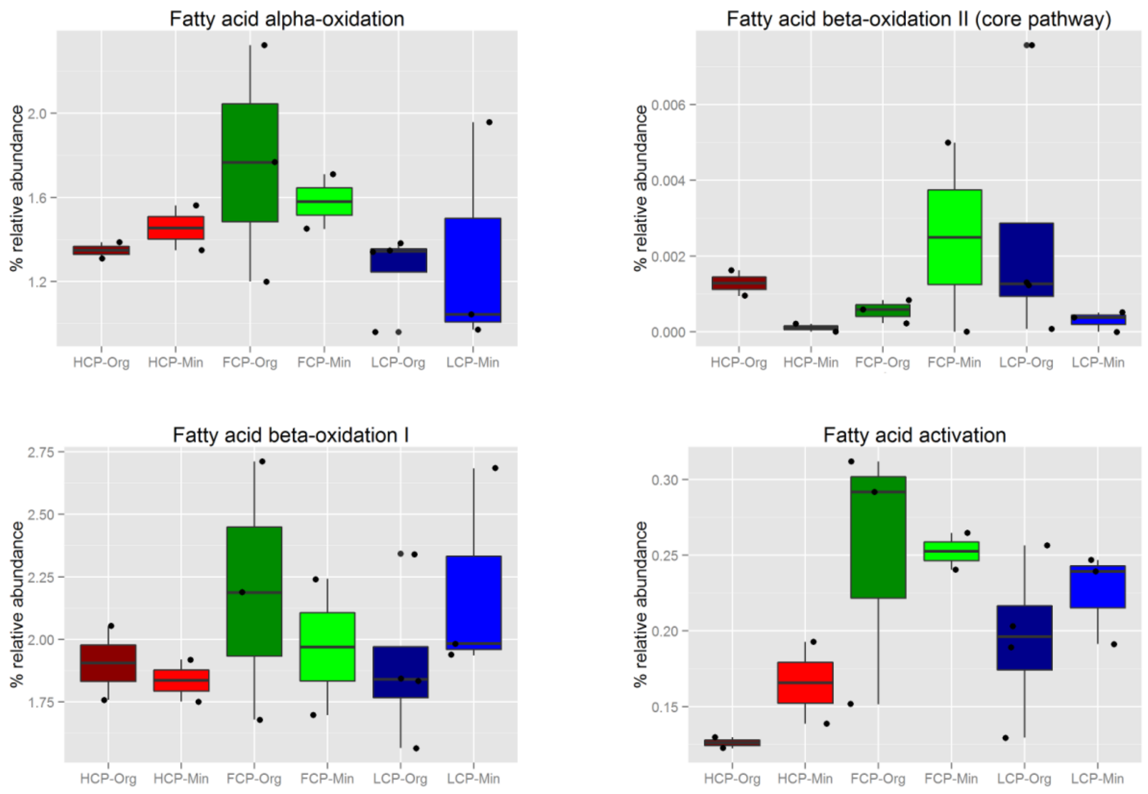

Supplementary Figure 18. Relative distribution of genes involved in fatty acid oxidation in Barrow metagenomes. Error bars represent the standard error.

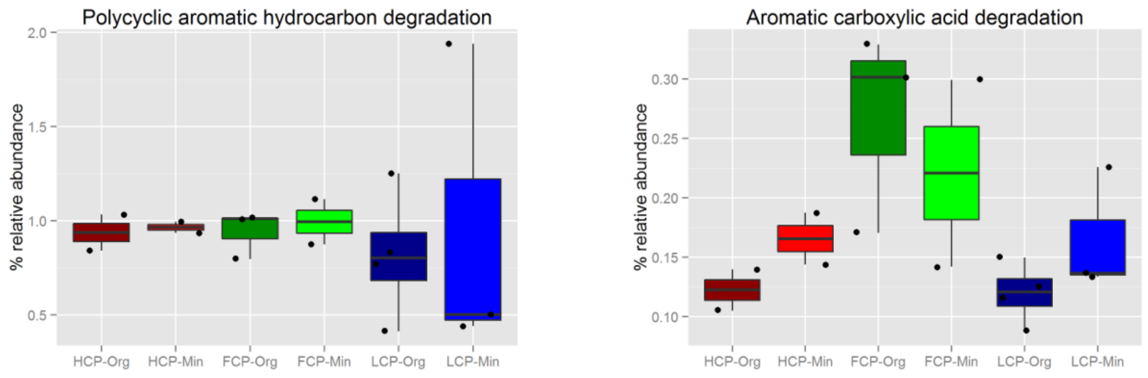

Supplementary Figure 19. Relative distribution of genes involved in hydrocarbon degradation in Barrow metagenomes. Error bars represent the standard error.

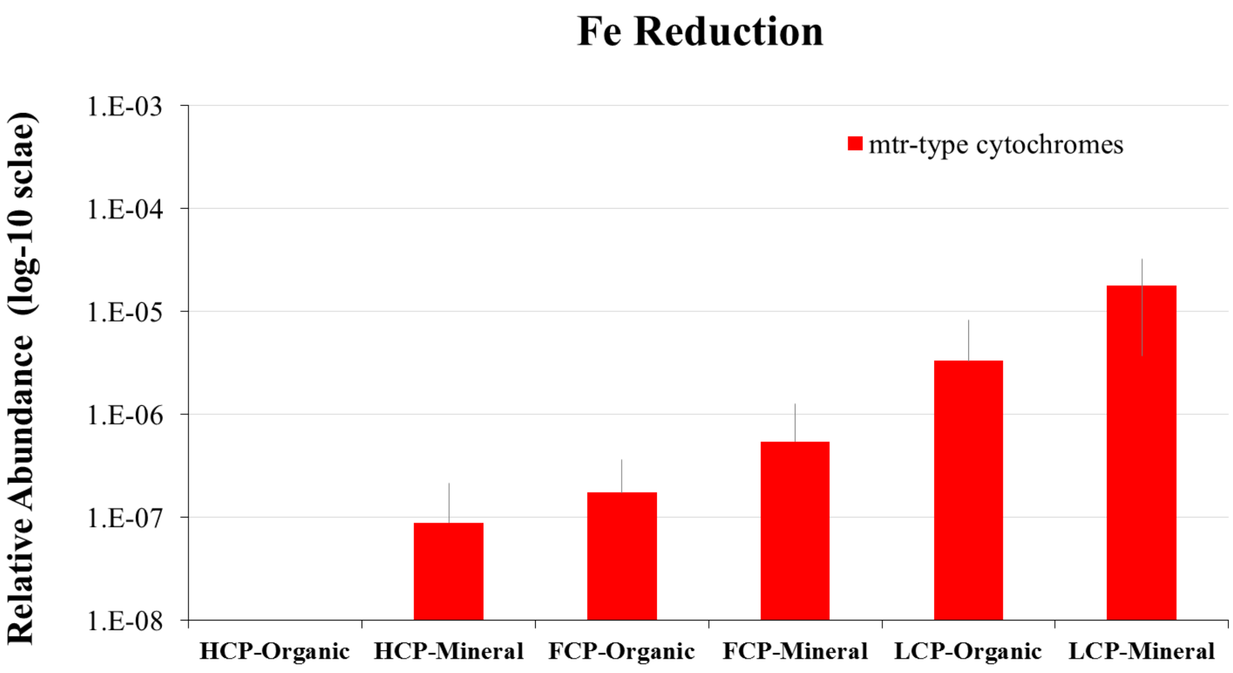

Supplementary Figure 20. Changes in the relative gene abundances of mtr-type decaheme cyctochromes in Barrow metagenomes were indicative of Fe-reduction potential in LC polygons. Error bars represent the standard error.

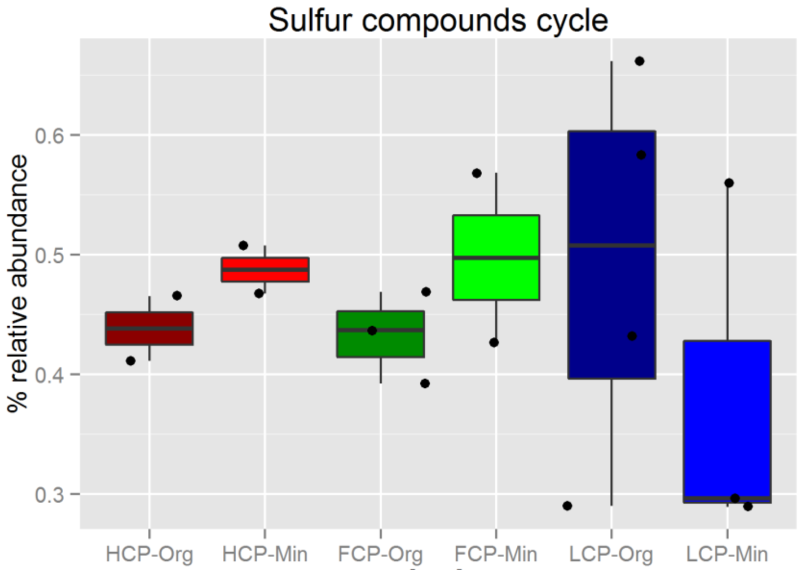

Supplementary Figure 21. Relative distribution of sulfur cycle genes in Barrow metagenomes. Error bars represent the standard error.

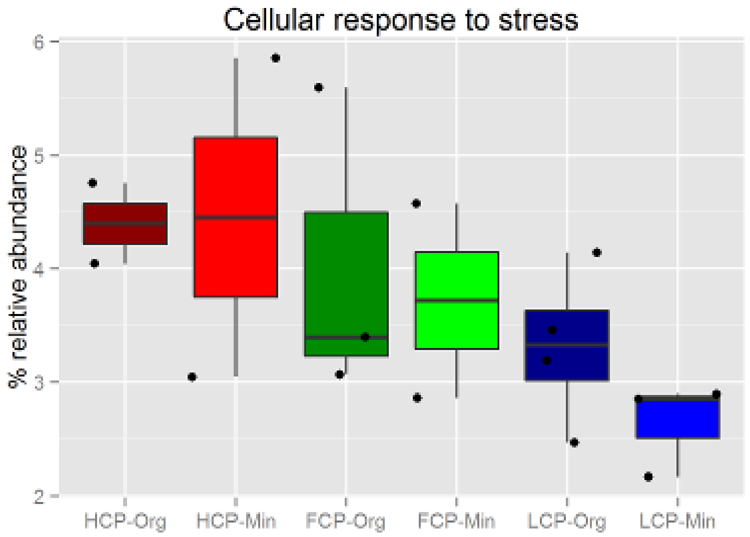

Supplementary Figure 22. Stress response genes were significantly higher in HC polygons in comparison with LC polygons. Error bars represent the standard error.

A)

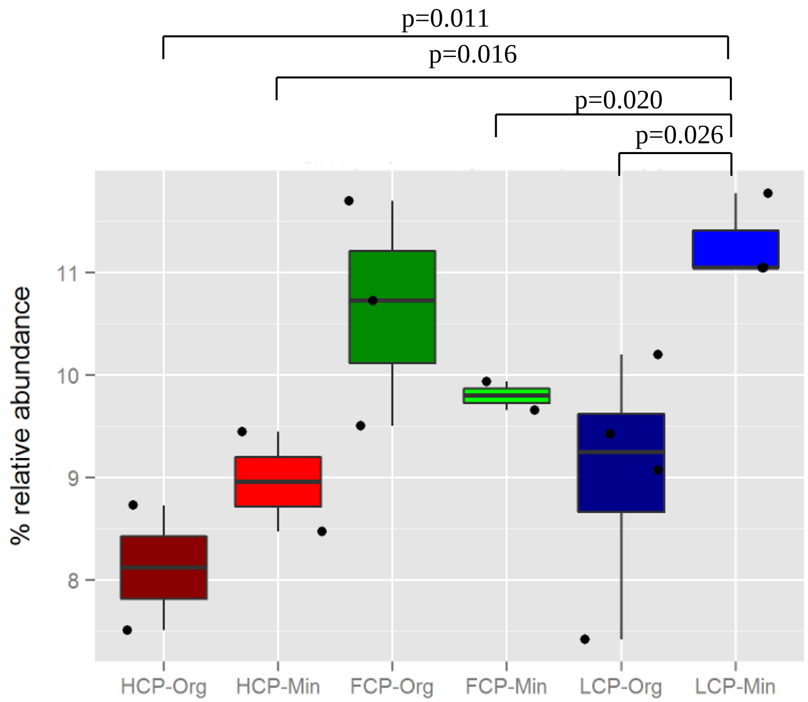

B)

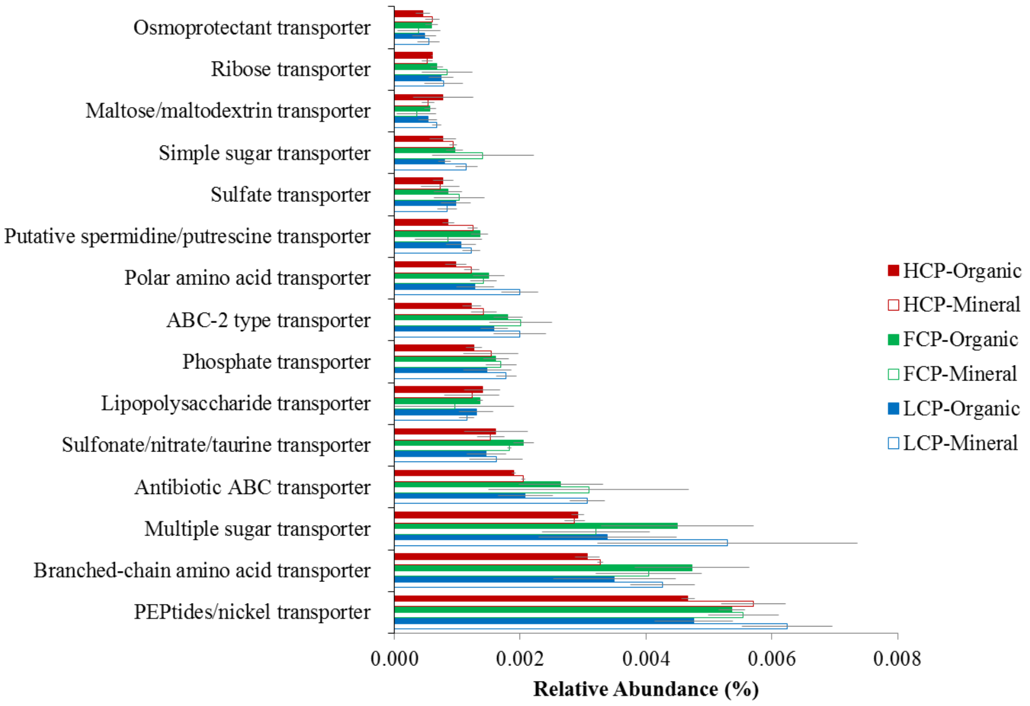

Supplementary Figure 23. A) Differences among ABC transporter genes in Barrow polygons. Error bars represent the standard error. B) Distribution of most abundant ABC type transporters in Barrow polygons
